# A selective cyclic integrin antagonist blocks the integrin receptors α_v_β_3 _and α_v_β_5 _and inhibits retinal pigment epithelium cell attachment, migration and invasion

**DOI:** 10.1186/1471-2415-5-16

**Published:** 2005-06-29

**Authors:** Stephan Hoffmann, Shikun He, Manlin Jin, Marianne Ehren, Peter Wiedemann, Stephen J Ryan, David R Hinton

**Affiliations:** 1 Doheny Eye Institute, Departments of Ophthalmology, Keck School of Medicine, University of Southern California 1355 San Pablo Street, Los Angeles 90033, CA, USA; 2 Department of Pathology, Keck School of Medicine, University of Southern California, 2011 Zonal Ave HMR 209, Los Angeles, CA 90033, USA; 3 Department of Ophthalmology, University of Leipzig, Liebigstrasse 10–14, 04103 Leipzig, Germany; 4 Berufsgenossenschaftliche Kliniken Bergmannsheil, University of Bochum, Department of Internal Medicine I, Bürkle-de-la-Camp-Platz 1, D-44789 Bochum, Germany

## Abstract

**Background:**

Proliferative vitreoretinopathy (PVR) is a leading cause of blindness after failed retinal reattachment surgery. PVR is characterized by the proliferation, migration and contraction of retinal pigmented epithelial cells (RPE), and these cellular responses are influenced by the expression and function of integrin receptors. The effect of a cyclic integrin antagonist containing the amino acid sequence Arg-Gly-Asp-D-Phe-Val (RGDfV), specific for the integrin receptors α_v_β_3 _and α_v_β_5_, was investigated on basic fibroblast growth factor (bFGF), platelet derived growth factor-BB (PDGF-BB), and serum induced human RPE proliferation, migration, invasion and attachment to the extracellular matrix. Furthermore, the effects of bFGF and PDGF-BB regulated expression of integrins α_v_β_3 _and α_v_β_5 _on RPE cells was examined.

**Methods:**

The effect of a cyclic integrin antagonist and a control peptide (0.01 μg/ml to 300 μg/ml) was investigated on serum or cytokine (bFGF or PDGF-BB pretreatment) induced human fetal RPE cell proliferation by H^3^-thymidine uptake. The effect of the cyclic integrin antagonist on RPE cell attachment onto different extracellular matrices (laminin, collagen IV, fibronectin), RPE cell invasion stimulated by PDGF-BB or serum, and migration stimulated by PDGF-BB, vascular endothelial growth factor (VEGF) or serum was explored. PDGF-BB and bFGF modulation of the integrin receptors α_v_β_3 _and α_v_β_5 _was evaluated by flow cytometry.

**Results:**

The integrin antagonist did not inhibit DNA synthesis stimulated by serum, bFGF, or PDGF-BB treatment. RPE attachment onto fibronectin was inhibited in a concentration range of 1–10 μg/ml (p < 0.05). Attachment of the RPE cells onto collagen IV and laminin was inhibited in a range of 3–10 μg/ml (p < 0.05). Serum and PDGF-BB stimulated migration was inhibited by the cyclic integrin antagonist in a concentration range of 1–10 μg/ml (p < 0.05). Furthermore, the cyclic integrin antagonist inhibited PDGF-BB stimulated RPE cell invasion through fibronectin (3μg/ml: 66% inhibition, p < 0.001). In each of these experiments, the control peptides had no significant effects. PDGF-BB and bFGF pretreatment of RPE cells increased the expression of integrin receptors α_v_β_3 _(bFGF: 1.9 fold, PDGF-BB: 2.3 fold) and α_v_β_5 _(bFGF: 2.9 fold, PDGF-BB: 1.5 fold).

**Conclusion:**

A selective inhibition of the integrin receptors α_v_β_3 _and α_v_β_5 _through a cyclic integrin antagonist is able to inhibit RPE cell attachment, migration and invasion. Since these steps are of importance for the progression of PVR, a cyclic integrin antagonist should be further evaluated for the treatment of this disease.

## Background

Integrins are a family of heterodimeric, non-covalently bound cell surface receptors, which mediate cell-cell and cell to extracellular matrix (ECM) adhesion. They are transmembrane glycoproteins consisting of a larger α and a smaller β subunit. In mammals, 18 α- and 8 β integrin genes encode polypeptides that combine to form 24 αβ heterodimeric receptors [1]. By mediating cell-cell and cell-matrix contact, the integrins are involved in a spectrum of physiologic and pathologic processes. Integrin expression is modulated by several cytokines, growth factors and the extracellular matrix in different cell types [2].

The integrins α_v_β_3 _and α_v_β_5 _have been shown to be of particular significance for the progression of neovascularization. Both integrin receptors contain RGD sequences containing the amino acid sequence Arg-Gly-Asp. Vitronectin receptor integrins of the α_v _– chain subfamily interact with their target proteins via the tripeptide sequence RGD (NH2-arginine-glycine-aspartic acid-COOH) [3,4]. The blockage of these RGD receptor sequences induces endothelial cell apoptosis by inhibiting their binding to the ECM [5,6]. In addition fibroblast attachment onto fibrinogen, a substep involved in wound healing, is inhibited by blockage of the α_v_β_3 _integrin receptor [7]. Therefore, the integrin α_v_β_3 _plays a critical role during wound repair as an adhesion receptor and as a signaling receptor [8]. As an adhesion receptor, it has the exceptional ability to bind vitronectin, fibronectin and fibrinogen, the three major proteins present in the wound provisional matrix [9].

In proliferative vitreoretinopathy (PVR), a disease of the posterior eye characteristically induced by trauma or failed retinal reattachment surgery, integrins are also of importance for the progression of the disease [10,11]. PVR, an exaggerated wound healing process, is characterized by the proliferation, migration and contraction of retinal pigment epithelial cells (RPE), fibroblasts, glial cells and macrophages. PVR membranes are formed on, or under the sensory retina; their contraction may lead to retinal detachment and blindness. RPE cells play a dominant role in the pathobiology of the disease [12]. Growth factors including basic fibroblast growth factor (bFGF), platelet derived growth factor-BB (PDGF-BB), and vascular endothelial growth factor (VEGF), and the ECM molecule fibronectin, regulate proliferation, migration and invasion of the RPE cell and are expressed in PVR membranes [12,13]. Fibronectin is an adhesive substrate for both the integrin receptors α_v_β_3 _and α_v_β_5 _[14]. Several studies have identified integrins α_v_β_3_, α_v_β_5_, integrin alpha subunits 2,3,4,5,6, V, and integrin beta subunits 1, 2, 3 on RPE cells and cells within PVR membranes [15-17]. The ECM-cell interactions are thought to be mediated largely through this family of cell surface receptors. Because cell-ECM interaction is important in the process of wound healing, one could predict that integrins would be actively involved in the pathogenesis of PVR. For PVR, it was shown *in vivo*, that tractional retinal detachment could be inhibited [11] by blockage of integrin-ECM binding by an RGD containing disintegrin.

Peptide antagonists specific for individual integrins have been developed, including cyclic RGD peptides selective for the α_v _integrin [18], the α_v_β_3 _integrin [19], and the α_v_β_5 _integrin [18-21]. Sequence alterations in the backbone of the RGD-containing cyclic peptides that result in conformational differences in the interatomic distance between the C_β _atoms of Arg and Asp, have been shown to correlate with selective peptide recognition [18,22,23].

In this study we investigated the modulatory effect of the cytokines PDGF-BB and bFGF on the expression of the RGD containing integrin receptors α_v_β_3 _and α_v_β_5 _on human fetal RPE cells. Furthermore, the effect of a specific cyclic integrin antagonist that blocks the integrin receptors α_v_β_3 _and α_v_β_5_, was investigated for its effects on RPE attachment, proliferation, migration and invasion. A specific cyclic integrin antagonist was chosen for its higher affinity and stronger binding capacity to the α_v_β_3 _and α_v_β_5 _integrins compared to a non-cyclic integrin antagonist [24,18].

## Methods

All cell culture solutions, media and ECM molecules (fibronectin, laminin and vitronectin) were purchased from Sigma (Sigma, St. Louis, MO).

### Isolation and culture of human RPE cells

Human RPE cells were isolated from fetal donor eyes (gestation time over 22 weeks) which were obtained from the Anatomic Gift foundation (Woodbine, GA) as has been described [25]. RPE cells were seeded onto laminin-coated 6 well plates (Fisher Scientific, Tustin, CA) using DMEM with 10 % fetal bovine serum, 1 % penicillin/streptomycin and glutamine. Cells from passage 3 to 5 were used in all experiments.

### Synthesis of the cyclic integrin antagonist and its control peptide

The cyclic integrin antagonist RGDfV, specific for the inhibition of integrins α_v_β_3 _and α_v_β_5 _[18-21] and the control peptide RADfV were synthesized as described [6] at the USC/ Norris Cancer Peptide synthesis facility. The peptides were dissolved in Hank's Balanced Salt Solution (HBSS), and stored at -80°C. HPLC analysis revealed a purity > 99% of the synthesized peptide.

### Flow cytometry

RPE cells (30,000 cells/well) were treated for 5 days with the cytokines bFGF and PDGF-BB at a concentration of 10 ng/ml in 6 well plates. Cells were detached, fixed with 4 % paraformaldehyde, blocked with 5 % goat serum for 30 minutes, centrifuged at 4°C, and incubated with a monoclonal mouse antibody against α_v_β_3 _or α_v_β_5 _(5 μg/ml) (Chemicon, CA) for one hour. After washing, a fluorescein conjugated secondary antibody (Chemicon, CA) directed against the mouse antibodies was added in a dilution of 1:100 for one hour. The expression of the integrin receptors α_v_β_3 _or α_v_β_5 _was measured using a fluorescent-activated cell sorter (FACStar plus, Becton Dickinson, Mountain View, CA). Forward and side light scatter was used to gate the desired scattered events (RPE cells) from dead cells and debris. The fluorescent index was determined by multiplying the percentage of cells by their mean fluorescence. At least 10,000 cells were evaluated/experiment and all experiments were performed in triplicate.

### Cell counting and viability studies

Subconfluent, bFGF (10 ng/ml) or PDGF-BB (10 ng/ml) pre-stimulated RPE cells were seeded at a density of 8 × 10^4 ^cells per well in 6 well plates containing DMEM with 10 % FBS and the cyclic integrin antagonist or the control peptide (0.01 μg/ml, 0.1 μg/ml, 1 μg/ml, 30 μg/ml and 300 μg/ml). The cells were exposed during the proliferation assay to 10% FBS or the cytokines bFGF and PDGF-BB (10 ng/ml). After 3 days of culture, the cell number was determined using a hemocytometer. The experiments were performed six times, each in triplicate. Cell viability was estimated by observation of cell morphology and by exclusion of 0.4 % trypan blue solution [26].

### ^3^H-thymidine uptake

RPE cells (10,000 cells/well) were seeded into DMEM with 10 % FBS in 24 well plates with the cyclic integrin antagonist or the control peptide (0.01 μg/ml, 0.1 μg/ml, 1 μg/ml, 30 μg/ml and 300 μg/ml). To determine a possible stronger effect of the cyclic integrin antagonist on RPE cell proliferation, cells were pre-stimulated for 5 days with bFGF (10 ng/ml) or PDGF-BB (10 ng/ml) (R&D Systems, Minneapolis, MN) to induce an upregulation of the integrin receptors α_v_β_3 _or α_v _β 5. After the pre-incubation, cells were seeded in DMEM with 10 % FBS, with the cyclic integrin antagonist or control peptide, and bFGF (10 ng/ml) or PDGF-BB (10 ng/ml). Cells were treated for three days with the peptides, then ^3^H-thymidine uptake was measured as described previously [25]. Results are presented as the percentage of control values.

### Migration assay

Migration was performed in a modified Boyden chamber (Falcon, Becton Dickinson Labware, Franklin Lakes, NJ). Subconfluent RPE cells, which have been pre-exposed to the cyclic integrin antagonist peptide (0.001 ng/ml, 0.01 ng/ml, 0.1 ng/ml, 1 ng/ml, 3 ng/ml and 10 ng/ml) or the control peptide (10 μg/ml) over night were seeded into DMEM with 1 % FBS into the upper part of a Boyden chamber (50,000 cells/chamber). The cyclic integrin antagonist was added directly to the upper part of the chamber (0.001 ng/ml, 0.01 ng/ml, 0.1 ng/ml, 1 ng/ml, 3 ng/ml, or 10 ng/ml). To evaluate nonspecific activity of the integrin antagonist, a control peptide (10 μg/ml) was used. The lower part of the chamber was filled with DMEM with 1 % FBS containing the chemoattractant PDGF-BB (10 ng/ml) or the ECM molecule fibronectin (30 ng/ml). RPE cells were incubated for 8 hours in a humidified CO_2_-incubator. Then the cells in the upper part were scraped away and the membrane was fixed with 1/2 strength Karnovsky's solution and stained with 0.25 % Richardson's solution. RPE cells on the lower side of the membrane were counted with the help of an inverted microscope and a grid (Carl Zeiss, Jena, Germany) in five random high power fields (200 X) in triplicate.

### Cell attachment

Ninety-six well plates were coated with the ECM molecules laminin, fibronectin or collagen IV (25 μg/ml). RPE cells were pre-incubated with the cyclic integrin antagonist (concentrations as above) or control peptide (10 μg/ml) over night. Then 15 × 10^3 ^cells, suspended in 100 μl DMEM with the cyclic or control peptide were seeded in each well and allowed to attach for 60 minutes. A tetrazolium MTT [3-(4,5-dimethylthiazolyl-2)-2,5-diphenyltetrazolium bromide] solution (20 μl/well) (Boehringer Mannheim, Indianapolis, IN, USA) was added for 5 hours of incubation. Then the supernatants were decanted, 150 μl of 100% DMSO added, and the cells were placed on a shaker for 10 minutes. Absorbance of the cells was measured at 550 nm with a Dynatech MR 600 spectrophotometric microplate reader.

### Invasion assay

RPE cell invasion was examined in a modified Boyden chambers. The insert membrane was coated overnight with fibronectin (25 μg/ml). Subconfluent RPE cells were pretreated overnight with the cyclic integrin antagonist (3 μg/ml) or the control peptide (3 μg/ml). An RPE cell suspension (50,000 cells/0.1 ml) in DMEM with 0.4 % FBS and 3 μg/ml cyclic integrin antagonist or control peptide was added into the upper part of the chamber. 600 μl DMEM with 0.4% FBS and PDGF-BB (10 μg/ml) was added into the lower part of the chamber. Invasion was measured with and without PDGF-BB stimulation after 5 hours at 37°C (95 % air/ 5 % Co_2_). After PBS washing and methanol fixation (10 minutes at 4°C), counterstaining with hematoxylin was performed. Cells in the upper chamber were wiped away. RPE cells that invaded the lower surface of the membrane were quantified by cell counting (320 × magnification).

### Statistical analysis

All experiments were repeated in triplicate. Standard deviations and averages were calculated. The data obtained were analyzed for significance using one way analysis of variance (ANOVA). Data with a p-value of less then 0.05 were considered to be statistically significant.

## Results

### The cyclic integrin antagonist does not inhibit RPE cell proliferation

The RPE cells were stimulated to incorporate thymidine by either 10% serum or by 10 ng/ml bFGF or 10 ng/ml PDGF-BB (p < 0.01). Neither the integrin antagonist, nor control peptide showed any significant effect on serum, PDGF-BB or bFGF stimulated DNA synthesis. (data not shown). In addition, cell counting showed no effects of the integrin antagonist or the control peptide on serum or cytokine stimulated proliferation. Furthermore, a lack of toxicity for the integrin antagonist and the control peptide in the concentration range tested (0.01 μg/ml, 0.1 μg/ml, 1 μg/ml, 30 μg/ml and 300 μg/ml) was determined by use of the trypan blue exclusion assay (data not shown).

### bFGF and PDGF-BB upregulate surface expression of α_v_β_3 _and α_v_β_5_

**Figure 1:**
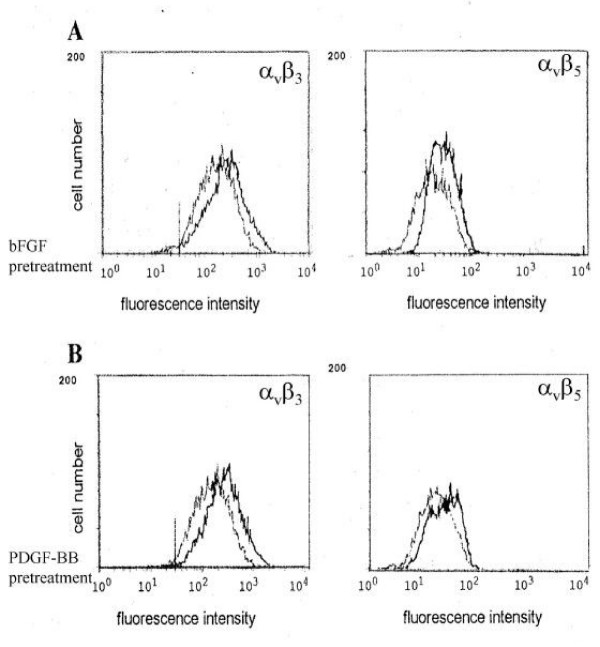
**A. **Expression of the integrin receptors α_v_β_3 _and α_v_β_5 _after pretreatment with the cytokine bFGF in a concentration of 10 ng/ml for 5 days.**B.** Expression of the integrin receptors α_v_β_3 _and α_v_β_5 _after pretreatment with the cytokine PDGF-BB in a concentration of 10 ng/ml for 5 days.

Pre-stimulation of RPE cells for 5 days with the cytokines PDGF-BB and bFGF (10 ng/ml) increased the expression of the integrin receptors α_v_β_3 _and α_v_β_5 _(Fig. 1; representative flow cytometric study). Incubation with 10 ng/ml bFGF upregulated the expression of α_v_β_3 _(1.9 fold) and strongly enhanced the expression of α_v_β_5 _(2.9 fold). PDGF-BB (10 ng/ml) strongly enhanced α_v_β_3 _(2.3 fold), and less strongly upregulated surface expression of α_v_β_5 _(1.5 fold).

### The cyclic integrin antagonist inhibits RPE attachment onto ECM

**Figure 2:**
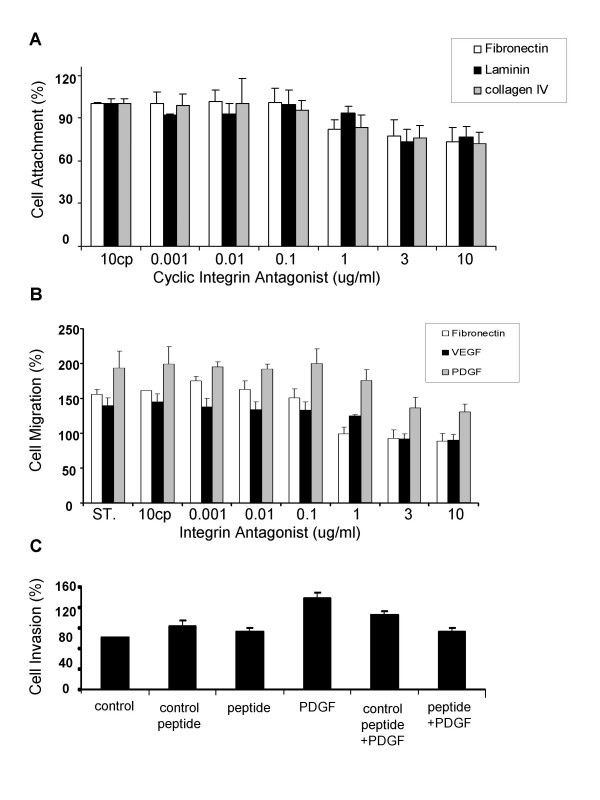
**A. **RPE attachment on fibronectin, laminin and collagen IV. The cells were pretreated before the assay with the cyclic integrin antagonist, or the control peptide (10 μg/ml). Untreated RPE cells were used in addition as a standard (ST). **B. **RPE cell migration induced by PDGF-BB, VEGF and fibronectin. The effect of the cyclic integrin antagonist or the control peptide (cp) on migration is shown. (ST = untreated RPE cells) **C. **Effect of the cyclic integrin antagonist, (3 ug/ml) or the control peptide on RPE invasion through fibronectin. Cell invasion through fibronectin (F) was stimulated with PDGF-BB (10 ng/ml). Nonspecific effects were excluded by the use of a cyclic control peptide. DMEM without PDGF-BB was used as a control in the assay.

RPE cell attachment onto fibronectin was inhibited significantly in the range of 1 to 10 μg/ml of cyclic integrin antagonist (p < 0.05) (Fig. 2A). A concentration of 10 μg/ml control peptide showed no effect. RPE cell attachment was inhibited 18 % with 1 μg/ml (p = 0.03), 23 % with 3 μg/ml (p < 0.005) and 27 % with 10 μg/ml (p < 0.001) onto fibronectin. An ID_50 _of 18.5 μg/ml cyclic integrin antagonist was calculated for the RPE cell attachment onto fibronectin. The attachment onto collagen IV was inhibited 25 % with 3 μg/ml (p < 0.001), and 29 % with 10 μg/ml (p < 0.001). Lower concentrations showed no significant effect. An ID_50 _of 17.2 μg/ml cyclic peptide was calculated for RPE cell attachment onto collagen IV. Attachment onto laminin was inhibited by the cyclic integrin antagonist in the range of 3–10 μg/ml (3 μg/ml, 27 % inhibition (p < 0.001); 10μg/ml: 23 % inhibition (p < 0.001). An ID_50 _of 21.7 μg/ml was calculated for the inhibition of RPE cell attachment onto laminin.

### The cyclic integrin antagonist inhibits RPE cell migration

The ECM component fibronectin (30 ng/ml) induced chemotaxis when placed as a soluble agent in the lower part of the Boyden chamber (Fig. 2B). This migratory response was inhibited by the cyclic integrin antagonist but not by the control peptide. RPE cell migration was inhibited in a concentration range of 1–10 μg/ml (1μg/ml, 18 % inhibition (p < 0.001); 3 μg/ml, 23 % inhibition (p < 0.001); 10 mg/ml, 27 % inhibition (p < 0.001)). An ID_50 _of 18.5 μg/ml integrin antagonist was calculated for the serum induced migration. The migratory response induced by PDGF-BB (10 ng/ml) (93 % stimulation of migration) was diminished by the cyclic integrin antagonist. (1μg/ml, 26 % inhibition (p < 0.001); 3 μg/ml, 66 % inhibition (p < 0.001); and 10μg/ml, 74 % inhibition (p < 0.001)). An ID_50 _of 5.8 μg/ml was calculated for the inhibition of PDGF-BB induced migration of RPE cells. VEGF induced a migratory response of RPE cells by 39 %. This migratory response was inhibited by the cyclic integrin antagonist in a concentration range of 3–10 μg/ml (p < 0.01).

### The cyclic integrin antagonist inhibits PDGF-BB stimulated invasion through fibronectin

PDGF-BB strongly stimulated RPE cell invasion (77 %, (p < 0.001)) through fibronectin coated onto the lower surface of a membrane insert in a modified Boyden chamber. This invasion was inhibited by the cyclic integrin antagonist (3 μg/ml, 66 % inhibition (p < 0.001)). The control peptide had no significant effect on invasion (Fig. 2C). An ID_50 _of 2.3 μg/ml was calculated for the effects of the cyclic integrin antagonist on PDGF-BB induced RPE invasion through fibronectin.

## Discussion

These data provide support for the suggestion that inhibition of the integrin receptors α_v_β_3 _and α_v_β_5 _by a specific cyclic integrin antagonist is feasible for inhibiting RPE cell attachment, migration and invasion and may serve as an adjunctive therapy for disorders such as PVR. Integrins have been shown to influence cell signaling pathways that promote cell proliferation in concert with cytokines [27]. Interestingly, our data show that cyclic integrin inhibition of α_v_β_3 _and α_v_β_5 _had no effect on serum, PDGF-BB or bFGF induced proliferation of RPE. Since we also show that bFGF and PDGF-BB upregulate both α_v_β_3 _and α_v_β_5_, and that proliferation is not inhibited in these stimulated cells, we can infer that the lack of effect of the cyclic peptide on proliferation is not due to lack of integrin expression. Consistent with the lack of effect on proliferation, we found that the cyclic integrin inhibitor did not induce significant cell death. This suggests that in the conditions studied here, the effect of integrin inhibition by the cyclic peptide on survival and proliferation of RPE cells may differ from previously described effects on endothelial cells [28], specific tumor cells [29] and mammary epithelial cells [30].

Cell attachment of RPE cells onto fibronectin, laminin and collagen IV was inhibited by the cyclic integrin antagonist. Because substrate attachment and interaction is important for activated RPE function [31], it is reasonable to predict that anti-adhesive therapy holds promise for the therapy of PVR. The cyclic integrin antagonist has been shown previously to inhibit specific ligand binding or cell adhesion events mediated by α_v_β_3 _or α_v_β_5 _[18]. Blockage of RPE attachment onto laminin, collagen IV and fibronectin is of specific interest, because of the strong expression of these matrix proteins in PVR [12]. The ECM fibronectin reacts with the α_v_β_3 _receptor with high specificity and affinity, supporting cell adhesion to this matrix protein. The α_v_β_5 _receptor binds in a similar way to fibronectin [32]. The blockage of α_v_β_3 _and α_v_β_5 _thus provides a reasonable explanation for the decreased attachment of RPE onto collagen IV, fibronectin and laminin in the presence of the inhibitory cyclic peptide.

RPE cell migration is another important process in the development of PVR. Without migration, RPE cells would not gain access to the vitreous and form vitreoretinal membranes. For RPE cells, it was shown previously that monoclonal antibody inhibition of integrin subunit β _1 _inhibits RPE cell migration from wound edges in organ culture [14]. Integrin signaling results in actin cytoskeleton polymerization, which in turn regulates cell shape and motility [33]. The α_v_β_3 _receptor appears to be involved in pathways that regulate intracellular pH and intracellular Ca^2+ ^[34] and may therefore be involved in migration in response to motility factors and extracellular matrix components [35]. The same range of concentration of the cyclic peptide inhibited migration induced by both soluble fibronectin and PDGF-BB. This result may in part be due to the finding that PDGF-BB induced migration is mediated by signaling pathways that are also used by integrin receptors [36-38].

The cyclic integrin antagonist had a particularly strong effect on RPE invasion into fibronectin. This may be due to the concurrent effects of the peptide on the inhibition of adhesion, migration and proteolytic activity since each of these steps may play a critical role in the invasion of the ECM.

The cyclic integrin antagonist described here blocks both α_v_β_3 _and α_v_β_5_. In contrast, highly selective integrin antagonists such as LM609 block only α_v_β_3_. Therefore, a stronger inhibitory effect of the cyclic integrin antagonist can be expected as has been shown in tumors [36]. While increased specificity could result in lower toxicity, we did not find that the cyclic integrin antagonist inhibited thymidine incorporation, or that it increased trypan blue uptake in the concentrations tested.

## Conclusion

The cyclic integrin antagonist RGDfV inhibits RPE migration, attachment and invasion, and should be further investigated as a potential adjuvant pharmacological treatment for PVR.

## Competing interests

The author(s) declare that they have no competing interests

## Authors' contributions

All authors have been involved in the experimental design, data collection and manuscript preparation. The final manuscript has been read and approved by all authors.

## Pre-publication history

The pre-publication history for this paper can be accessed here:
